# Adaptive Immune Responses in Primary Cutaneous Sarcoidosis

**DOI:** 10.1155/2011/235142

**Published:** 2011-03-30

**Authors:** Matteo Bordignon, Paola Rottoli, Carlo Agostini, Mauro Alaibac

**Affiliations:** ^1^Unit of Dermatology, University of Padua, Via Cesare Battisti 206, 35128 Padua, Italy; ^2^Respiratory Diseases Section, Department of Clinical Medicine and Immunological Sciences, Siena University, 53100 Siena, Italy; ^3^Department of Clinical and Experimental Medicine, University of Padua, Via Cesare Battisti 206, 35128 Padua, Italy

## Abstract

Sarcoidosis is a multisystemic inflammatory disorder with cutaneous lesions present in about one-quarter of the patients. Cutaneous lesions have been classified as specific and nonspecific, depending on the presence of nonnecrotizing epithelial cell granulomas on histologic studies. The development and progression of specific cutaneous sarcoidosis involves a complex interaction between cells of the adaptive immune systems, notably T-lymphocytes and dendritic cells. In this paper, we will discuss the role of T-cells and skin dendritic cells in the development of primary cutaneous sarcoidosis and comment on the potential antigenic stimuli that may account for the development of the immunological response. We will further explore the contributions of selected cytokines to the immunopathological process. The knowledge of the adaptive immunological mechanisms operative in cutaneous sarcoidosis may subsequently be useful for identifying prevention and treatment strategies of systemic sarcoidosis.

## 1. Introduction

Sarcoidosis is a multisystemic inflammatory disorder characterized by the accumulation of mononuclear phagocytes with the formation of nonnecrotizing epithelial cell granulomas. Multiple organs may be involved, including lungs, mediastinal and peripheral lymph nodes, liver, spleen, skin, eyes, and parotid glands; central nervous system, heart, upper respiratory tract, bones, and joints are less frequently but usually more severely involved [1, 2]. 

Sarcoidosis is characterized by local immune hyperactivation associated with clinical anergy [3]. The pathogenesis of sarcoidosis is suspected to be a host immunologic response to an antigenic exposure [4]. The role of T-lymphocytes in the recognition of specific antigens and in the amplification of inflammatory responses has been well established [5]. In addition, dendritic cells have recently been shown to have a prominent role in the immunopathological processes operating in this condition [6, 7]. 

Cutaneous involvement in sarcoidosis occurs in about one-quarter of the patients and is generally observed at the onset of the disease process although it may occur coincident with or after systemic involvement [8, 9]. Therefore, cutaneous lesions can be an initial presentation and are probably an important factor in the investigation of the etiology of sarcoidosis. 

Skin lesions may be classified in specific, when histology shows typical noncaseating granulomatous inflammation, or non specific, in presence of reactive process without granulomas. 

## 2. Clinical Aspects of Primary Cutaneous Sarcoidosis

The frequency of specific skin involvement ranges from 9% to 37% [10]. All specific cutaneous lesions exhibit noncaseating granulomas on biopsy. Histological findings in specific sarcoid lesions show aggregates of epithelioid histiocytes with occasional Langhans giant cells and few or no other inflammatory cells, the so-called naked or sarcoidal noncaseating granulomas. Frequently, there are inclusion bodies in giant cells [11]. The centre of granulomas is typically surrounded by CD4+ lymphocytes, rare CD8+ lymphocytes and mature macrophages. Despite the same histologic appearance, clinical manifestations of primary cutaneous sarcoidosis may be variable. 

The most common types of specific skin manifestations are maculopapular lesions. They commonly appear on the face with a purple or red-brown appearance but may also be observed on lips, neck, upper trunk, extremities, and rarely mouth; these lesions show typical apple-jelly colour when examined by diascopy [10].

 Plaques are larger, red-brown, infiltrated lesions that are present on face, scalp, shoulders, arms, and buttocks. The lesions may be single or multiple and are associated with chronic course of disease. When plaques are multiple, distribution of the lesions tends to be symmetric. They may be associated with large telangiectatic vessels or may exhibit thick scaling [12]. 

Specific cutaneous lesions of sarcoidosis may take form of mobil and indolent subcutaneous nodules that appear generally late in the course of the disease. The patients may present single or multiple nodules with a diameter between 0.5 and 2 cm without clinical alteration of the epidermal compartment. These nodules may be associated with sarcoidal involvement of lung, spleen, and liver [13].

Scar sarcoidosis is characterised by the development of red-purple infiltrated lesions at the site of previous scar; this phenomenon, of unknown etiology, may preced the onset of lung involvement or be simultaneously with systemic sarcoidosis [14].

Lupus pernio is characterized by an indolent, infiltrated red-brown or purple shiny plaque on nose, lips, cheeks, and ears, more frequent in African American women. Lupus pernio can be followed or be associated with chronic fibrotic disease, notably chronic fibrotic sarcoidosis of upper respiratory tract, lung fibrosis, chronic uveitis, and bone cystis [15]. 

## 3. Kveim-Siltzbach Reaction: An Immunological Model of Primary Cutaneous Sarcoidosis

The immunopathogenesis of primary cutaneous sarcoidosis has been studied by means of the Kveim-Siltzbach reaction. This is an accepted immunological model of sarcoidosis which is consistent with the hypothesis that this condition is determined by an amplified adaptive immune response to an exogenous antigen at sites of granuloma formation [16]. The Kveim-Siltzbach test consists of an intradermal injection of a suspension of human sarcoid tissue prepared from spleen and lymph node which leads to granuloma formation virtually identical to that of primary cutaneous sarcoidosis [17]. In contrast to a delayed-type hypersensitivity reaction, the development of a papular lesion at the injection site is observed 3 to 6 weeks later. This lesion shows histological changes of typical noncaseating granulomatous inflammation [16]. This method of testing has been abandoned, but the several studies carried out on this peculiar skin reaction have allowed a better understanding of disease pathogenesis. In particular, Kataria and Park studied the Kveim-Siltzbach test using sequential biopsies at varying intervals in the site of reaction [18]. After 6 hours from the intradermal injection, necrotic collagen associated with a perivascular infiltrate mainly composed of CD4+ T-lymphocytes could be observed. After 48 hours an increased number of monocyte was observed; subsequently, these cells tended to disappear probably after transformation into multinucleated giant cells and epithelioid cells. From day 12 to day 34, lymphocyte CD4+ T-cells continued to increase whereas the first Langhans giant cells were seen on day 12. Biopsy specimens taken on day 21, day 28, and day 34 demonstrated progressive infiltration of T-lymphocytes whereas B cells, monocytes, and immunoglobulin deposition were virtually absent [18]. It has been suggested that initial collagen necrosis is responsible of immobilisation of the putative antigen which may promote the development of sarcoid granulomas. The initial influx of lymphocytes and monocytes observed in sarcoid granulomas supports the hypothesis that antigen presentation is the initiating stimulus for granuloma formation [19]. The lack of a significant presence of B lymphocytes in the infiltrate of cutaneous sarcoidosis has been recently confirmed [20] and may support the view that the granulomatous inflammation observed in cutaneous sarcoidosis is essentially a cell-mediated process against an unknown antigen encountered through the epidermis [21]. However, a role of B cells activated by helper T-cells may not completely be excluded in light of the apparent therapeutic benefits of B cell depleting therapy in sarcoidosis [22].

## 4. Dendritic Cells in Primary Cutaneous Sarcoidosis

The Kveim-Siltzbach reaction is consistent with the view that in cutaneous sarcoidosis the antigen seems to pass through the epidermis. In support of this hypothesis, there are the recent findings concerning the distribution of dendritic cells in cutaneous sarcoidosis. 

Immune cells of the epidermis and dermis participate in the defense against pathogens, in particular through a network of cutaneous dendritic cells, which have a key role in initiating both innate and adaptive immune responses [23]. Two main populations of dendritic cells occur in normal skin: epidermal Langerhans cells (LCs) and dermal dendritic cells. LCs are involved in monitoring the epidermal microenvironment by taking up antigen and processing it into fragments that can be recognized by naive and effectors cells of the adaptive immune response. LCs are capable of antigen uptake and processing, but at this stage of differentiation they are unable to stimulate naive T-cells and therefore, are considered immature antigen presenting cells. After capture and antigen-processing LCs migrate out of the epidermis and “mature” their ability for acting as professional antigen presenting cells [24]. Dermal dendritic cells are mature antigen presenting cells which share several functional and phenotypic characteristics with LCs including the ability to activate both naive and effector T-cells [25].

It has been demonstrated an increased number of epidermal LCs in primary cutaneous sarcoidosis [25]. This phenomenon is particularly evident in the epidermis overlying sarcoidal granulomas and is probably related to the ability of these cells to capture an environmental antigens through the epidermal compartment [26]. Furthermore, a maturation step of LCs has been demonstrated in the subepidermal layer over the cutaneous sarcoid granuloma suggesting that immature LCs might migrate in the epidermis, where they become mature antigen presenting cells for T-lymphocytes [26]. In support of this hypothesis, there is the demonstration that mature dendritic cells are increased in cutaneous sarcoidosis and observed around the granuloma attached to T-lymphocytes [26]. 

**Figure 1:**
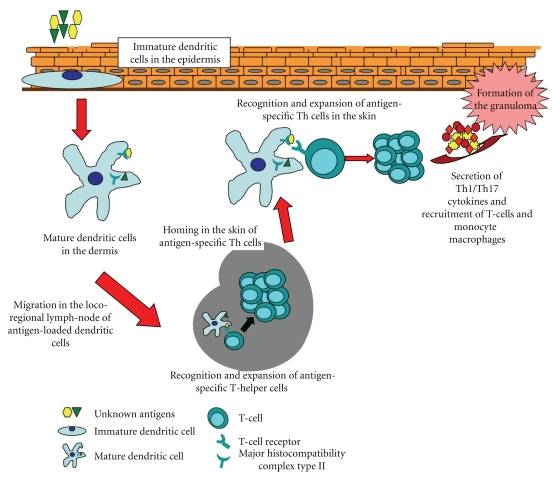
The basic immunopathological mechanisms that can be expected to be operative in primary cutaneous sarcoidosis.

The proposed immunological pathway (Figure 1) may also represent a potential mechanism for explaining systemic involvement following primary cutaneous sarcoidosis. In this context, an exogenous antigen penetrated through the skin is captured and processed by immature Langerhans cells. The subsequent systemic involvement of sarcoidosis could be determined by the spread through lymphatic vessels of antigen-bearing mature dendritic cells. This concept is supported by the clinical observation that specific cutaneous lesions in the course of sarcoidosis are generally observed before the onset of systemic disease [9]. 

## 5. Adaptive Immune Response in Response to a Specific Antigen in Primary Sarcoidosis

The nature of the antigens captured through the epidermis by LCs is unknown. Identification of these antigens would have importance for our understanding of cutaneous sarcoidosis and, possibly, cutaneous and systemic disease prevention. 

The specificity of the adaptive immune response resides in the antigen receptors on T and B cells [27]. These receptors are the results of a gene rearrangement process during lymphocyte maturation. The receptors produced by each lymphocyte have a unique antigen specificity which is determined by the structure of antigen binding site. This ensures that the receptor on each lymphocyte is single and specific. As a consequence, the adaptive immune response against a particular antigen is characterized by the initial stimulation of only a very small proportion of lymphocytes whose surface antigen receptors are able to recognize the specific antigen. The resulting antigen-driven proliferation results in the generation of large numbers of lymphocytes bearing the same antigen receptor and, consequently, the same antigen specificity [27]. 

It has been hypothesized that antigen specific T-cell clones are responsible for the onset and maintenance of cutaneous sarcoidosis [28]. In order to evaluate whether specific antigen-driven T-cell responses are involved in the initiation and/or maintenance of cutaneous sarcoidosis, it is necessary to examine whether T-cells infiltrating cutaneous sarcoid granulomas contain expanded clones of T-cells. Characterization of the transcripts of T-cell antigen receptor genes can be used to determine the clonality of T-lymphocytes populations infiltrating cutaneous sarcoidosis and may allow determination of their antigen specificity. These antigens may play a key role in the pathogenesis of cutaneous sarcoidosis. To this regard, an oligoclonal expansion of T-cells with a preferential expression of antigen T-cell receptor genes has been demonstrated at sites of Kveim-Siltzbach skin reactions giving evidence of an antigen-driven T-cell immune response [29]. A similar phenomenon was observed in patients with primary cutaneous sarcoidosis where an antigen-driven oligoclonal expansion of T-cells was detected [30]. However, the T-cell receptor gene usage in T-cell clones from patients with primary cutaneous sarcoidosis showed interindividual variability, thus indicating that the inappropriate response observed in cutaneous sarcoidosis is probably not determined by a common antigenic stimulus, but it may be the result of an individual predisposition in responding to a variety of self/nonself antigens [30]. 

## 6. The Role of Cytokines in Cutaneous Sarcoidosis

Cytokines are extracellular signalling proteins that mediate the effector functions of a variety of inflammatory cells. In particular, cytokine secretion by T lymphocytes has a key role in the adaptive immune responses against pathogens and/or autoantigens. On the basis of cytokine secretion, T helper (Th) lymphocytes are divided into at least two distinct subsets: type 1 helper T-cells (Th1 cells) and type 2 helper T-cells (Th2 cells). Th1 cells produce cytokines which mediate cell-mediated immunity, while cytokines produced by Th2 cells are associated with helper function for antibody production by B cells. Th1 cells are characterized by the production of interferons, tumor-necrosis factors, and interleukin-2, whereas a Th2 cytokine profile is associated with production of IL-4, IL-5, IL-6, IL-9, and IL-10 [31].

A Th1 predominant profile of cytokine production has been observed in systemic sarcoidosis, where only low levels of Th2-associated mediators have been detected [32, 33]. It is likely that a similar cytokine imbalance may be responsible for the onset and/or maintenance of cutaneous sarcoidosis. Although to date no studies have investigated the expression of these cytokines in cutaneous sarcoidosis, there is clinical evidence indicating a role for some cytokines associated with a Th1 immune response, notably interferon (IFN) alpha and tumor necrosis factor (TNF) alpha. 

Interferons are proinflammatory cytokines divided into two main classes: type I IFN such as IFN-alpha and type II IFN, notably IFN-gamma, both of which function as a bridge linking innate and adaptive immune responses and are responsible in shaping the subsequent development of adaptive immunity [34]. To this regard, several lines of evidence support the importance of IFN-alpha in regulation of the Th1 subset differentiation [35]. IFN-alpha, as other interferons, has antiviral and antioncogenic effects and, in combination with ribavirin, has become the current standard therapy for treating chronic hepatitis C patients [36]. The development of primary cutaneous as well as systemic sarcoidosis has been well-documented in chronic hepatitis C patients treated with IFN-alpha [37–41]. Furthermore, cutaneous sarcoidosis has also been observed during IFN-alpha based therapy for melanoma [42–44]. IFN-alpha induces T-cells to produce large amounts of Th1 cytokines and limited amounts of Th2 cytokines, favouring the development and enhancement of Th1-mediated responses. In particular, IFN-alpha induces STAT1 expression, a Th1 transcription factor that regulates several genes involved in the inflammatory response [45]. In this regard, a recent investigation has demonstrated increased STAT1 expression in sarcoidosis granulomas [46]. Taken together these data are consistent with the view that the enhancement of cell-mediated immunity by IFN-alpha in the presence of an as yet unknown cutaneous antigenic stimulus may be responsible for the development of cutaneous sarcoidosis in patients treated with this biological agent. 

TNF-alpha is a pro-inflammatory cytokine that plays a significant role in antigen-stimulated cell-mediated immune responses and in the development of Th1 responses [47]. In sarcoidosis, TNF-alpha participates in the induction and maintenance of granulomas and high levels of TNF-alpha seems to correlate with systemic disease progression [48]. There is clinical evidence that inhibition of TNF-alpha is an effective therapy for treating both systemic and cutaneous sarcoidosis which is consistent the view that TNF-alpha plays a prominent role in the inflammatory process seen in these conditions [49–53]. On the other hand, anti-TNF-alpha therapies can induce sarcoidosis, including primary cutaneous sarcoidosis [54, 55]. This may be determined by a paradoxical enhanced Th1 response with alteration of the cutaneous cytokine environment and enhancement of inflammatory cell infiltration into the skin. In particular, a cross-regulation between IFN-alpha and TNF-alpha has been recently proposed, where both cytokines may interact each other to affect the inflammatory responses [56]. According to this hypothesis, the paradoxical adverse effects of anti-TNF-alpha treatment, such as the exacerbation of psoriatic skin lesions in patients with psoriatic arthritis receiving anti-TNF-alpha therapy, can be considered as a consequence of the disbalance between the two cytokines. In this regard, it has been demonstrated that patients treated with TNF antagonists demonstrate upregulation of IFN-*α*-responsive genes [57]. The development of cutaneous sarcoidosis after TNF-blockers therapy may probably be triggered by an initial exposure to a skin antigen and it has been suggested that *Propionibacterium acnes* may promote the skin immune response observed in sarcoidosis [58]. Furthermore, TNF blockers increase the risk of infectious complications, including *Propionibacterium acnes *infection [59]. Taken together, these observations are consistent with the hypothesis, that both increased risk of skin infections and alterations in the skin immunological microenvironment may be responsible for the paradoxical occurrence of cutaneous sarcoidosis during treatment with TNF-blockers.

Interleukin-17 (IL17) is a proinflammatory cytokine that is involved in the pathogenesis of several chronic inflammatory diseases by upregulating the expression of various cytokines, chemokines, and cell-adhesion molecules [60]. T-helper 17 (Th17) cells are a recently discovered subset of CD4(+) helper T-cell that release large amounts of IL17 [60]. It has been demonstrated that this cytokine is involved in the alveolitic/granuloma phase of the disease and it seems plausible that it may also play a role in cutaneous granuloma formation [61]. 

## 7. Conclusions

Cutaneous sarcoidosis is a primary skin disorder with a poorly understood pathophysiologic mechanism. CD4+ lymphocytes and cutaneous dendritic cells probably represent the predominant cellular elements involved in the inflammatory responses. The adaptive immune system appears to actively participate in disease development and maintenance by elaboration of Th1 cytokines. Identification of the antigen(s) responsible for the adaptive immune activation may represent an important step in the recognition of the immunopathological mechanism operating in cutaneous sarcoidosis. The knowledge of the adaptive immunological mechanisms operative in cutaneous sarcoidosis may subsequently be useful for identifying prevention and treatment strategies for systemic sarcoidosis. 

## References

[1] Giuffrida TJ, Kerdel FA (2002). Sarcoidosis. *Dermatologic Clinics*.

[2] Iannuzzi MC, Rybicki BA, Teirstein AS (2007). Sarcoidosis. *New England Journal of Medicine*.

[3] Miyara M, Amoura Z, Parizot C (2006). The immune paradox of sarcoidosis and regulatory T cells. *Journal of Experimental Medicine*.

[4] Baughman RP, Lower EE, Du Bois RM (2003). Sarcoidosis. *Lancet*.

[5] Zissel G, Prasse A, Müller-Quernheim J (2010). Immunologic response of sarcoidosis. *Seminars in Respiratory and Critical Care Medicine*.

[6] Ota M, Amakawa R, Uehira K (2004). Involvement of dendritic cells in sarcoidosis. *Thorax*.

[7] Zaba LC, Smith GP, Sanchez M, Prystowsky SD (2010). Dendritic cells in the pathogenesis of sarcoidosis. *American Journal of Respiratory Cell and Molecular Biology*.

[8] Tchernev G (2006). Cutaneous sarcoidosis: the “great imitator”: etiopathogenesis, morphology, differential diagnosis, and clinical management. *American Journal of Clinical Dermatology*.

[9] Lodha S, Sanchez M, Prystowsky S (2009). Sarcoidosis of the skin: a review for the pulmonologist. *Chest*.

[10] English JC, Patel PJ, Greer KE (2001). Sarcoidosis. *Journal of the American Academy of Dermatology*.

[11] Ball NJ, Kho GT, Martinka M (2004). The histologic spectrum of cutaneous sarcoidosis: a study of twenty-eight cases. *Journal of Cutaneous Pathology*.

[12] Katta R (2002). Cutaneous sarcoidosis: a dermatologic masquerader. *American Family Physician*.

[13] Ahmed I, Harshad SR (2006). Subcutaneous sarcoidosis: is it a specific subset of cutaneous sarcoidosis frequently associated with systemic disease?. *Journal of the American Academy of Dermatology*.

[14] Selim A, Ehrsam E, Atassi MB, Khachemoune A (2006). Scar sarcoidosis: a case report and brief review. *Cutis*.

[15] Sharma OP, Papanikolaou IC (2009). Lupus pernio: a tale of four characters in search of a malady. *Sarcoidosis Vasculitis and Diffuse Lung Diseases*.

[16] James DG, Williams WJ (1991). Kveim-Siltzbach test revisited. *Sarcoidosis*.

[17] Richter E, Kataria YP, Zissel G, Homolka J, Schlaak M, Müller-Quernheim J (1999). Analysis of the Kveim-Siltzbach test reagent for bacterial DNA. *American Journal of Respiratory and Critical Care Medicine*.

[18] Kataria YP, Park HK (1986). Dynamics and mechanism of the sarcoidal granuloma: detecting T cell subsets, non-T cells, and immunoglobulins in biopsies at varying intervals of Kveim-Siltzbach test sites. *Annals of the New York Academy of Sciences*.

[19] Kataria YP, Holter JF (1997). Immunology of sarcoidosis. *Clinics in Chest Medicine*.

[20] De Jager M, Blokx W, Warris A (2008). Immunohistochemical features of cutaneous granulomas in primary immunodeficiency disorders: a comparison with cutaneous sarcoidosis. *Journal of Cutaneous Pathology*.

[21] Noor A, Knox KS (2007). Immunopathogenesis of sarcoidosis. *Clinics in Dermatology*.

[22] Belkhou A, Younsi R, El Bouchti I, El Hassani S (2008). Rituximab as a treatment alternative in sarcoidosis. *Joint Bone Spine*.

[23] Heath WR, Carbone FR (2009). Dendritic cell subsets in primary and secondary T cell responses at body surfaces. *Nature Immunology*.

[24] Nestle FO, Di Meglio P, Qin JZ, Nickoloff BJ (2009). Skin immune sentinels in health and disease. *Nature Reviews Immunology*.

[25] Martin AG, Kleinhenz ME, Elmets CA (1986). Immunohistologic identification of antigen-presenting cells in cutaneous sarcoidosis. *Journal of Investigative Dermatology*.

[26] Kurata A, Terado Y, Izumi M, Fujioka Y, Franke FE (2010). Where does the antigen of cutaneous sarcoidosis come from?. *Journal of Cutaneous Pathology*.

[27] Bonilla FA, Oettgen HC (2010). Adaptive immunity. *Journal of Allergy and Clinical Immunology*.

[28] Gerke AK, Hunninghake G (2008). The immunology of sarcoidosis. *Clinics in Chest Medicine*.

[29] Klein JT, Horn TD, Forman JD, Silver RF, Teirstein AS, Moller DR (1995). Selection of oligoclonal V*β*-specific T cells in the intradermal response to Kveim-Siltzbach reagent in individuals with sarcoidosis. *Journal of Immunology*.

[30] Mempel M, Flageul B, Suarez F (2000). Comparison of the T cell patterns in leprous and cutaneous sarcoid granulomas: presence of V*α*24-invariant natural killer T cells in T-cell-reactive leprosy together with a highly biased T cell receptor V*α* repertoire. *American Journal of Pathology*.

[31] Singh VK, Mehrotra S, Agarwal SS (1999). The paradigm of Th1 and Th2 cytokines: its relevance to aotoimmunity and allergy. *Immunologic Research*.

[32] Agostini C, Meneghin A, Semenzato G (2002). T-lymphocytes and cytokines in sarcoidosis. *Current Opinion in Pulmonary Medicine*.

[33] Gurrieri C, Bortoli M, Brunetta E, Piazza F, Agostini C (2005). Cytokines, chemokines and other biomolecular markers in sarcoidosis. *Sarcoidosis Vasculitis and Diffuse Lung Diseases*.

[34] Pestka S (2007). The interferons: 50 years after their discovery, there is much more to learn. *Journal of Biological Chemistry*.

[35] Pestka S, Krause CD, Walter MR (2004). Interferons, interferon-like cytokines, and their receptors. *Immunological Reviews*.

[36] Aronsohn A, Reau N (2009). Long-term outcomes after treatment with interferon and ribavirin in HCV patients. *Journal of Clinical Gastroenterology*.

[37] Tortorella C, Napoli N, Panella E, Antonaci A, Gentile A, Antonaci S (2004). Asymptomatic systemic sarcoidosis arising 5 years after IFN-*α* treatment for chronic hepatitis C: a new challenge for clinicians. *Journal of Interferon and Cytokine Research*.

[38] Daïen CI, Monnier A, Claudepierre P (2009). Sarcoid-like granulomatosis in patients treated with tumor necrosis factor blockers: 10 cases. *Rheumatology*.

[39] Neglia V, Sookoian S, Herrera M (2001). Development of cutaneous sarcoidosis in a patient with chronic hepatitis C treated with interferon alpha 2b. *Journal of Cutaneous Medicine and Surgery*.

[40] Rogers CJ, Romagosa R, Vincek V (2004). Cutaneous sarcoidosis associated with pegylated interferon alfa and ribavirin therapy in a patient with chronic hepatitis C. *Journal of the American Academy of Dermatology*.

[41] Fantini F, Padalino C, Gualdi G, Monari P, Giannetti A (2009). Cutaneous lesions as initial signs of interferon *α*-induced sarcoidosis: report of three new cases and review of the literature. *Dermatologic Therapy*.

[42] Alonso-Pérez A, Ballestero-Díez M, Fraga J, García-Díez A, Fernández-Herrera J (2006). Cutaneous sarcoidosis by interferon therapy in a patient with melanoma. *Journal of the European Academy of Dermatology and Venereology*.

[43] Pelletier F, Manzoni P, Jacoulet P, Humbert P, Aubin F (2007). Pulmonary and cutaneous sarcoidosis associated with interferon therapy for melanoma. *Cutis*.

[44] Suárez-García C, Pérez-Gil A, Pereira-Gallardo S (2009). Interferon-induced cutaneous sarcoidosis in melanoma. *Melanoma Research*.

[45] Chen H, Wang LW, Huang YQ, Gong ZJ (2010). Interferon-alpha induces high expression of APOBEC3G and STAT-1 in Vitro and in Vivo. *International Journal of Molecular Sciences*.

[46] Rosenbaum JT, Pasadhika S, Crouser ED (2009). Hypothesis: sarcoidosis is a STAT1-mediated disease. *Clinical Immunology*.

[47] Bazzoni F, Beutler B (1996). The tumor necrosis factor ligand and receptor families. *New England Journal of Medicine*.

[48] Baughman RP, Iannuzzi M (2003). Tumour necrosis factor in sarcoidosis and its potential for targeted therapy. *BioDrugs*.

[49] Baughman RP, Lower EE, Drent M (2008). Inhibitors of tumor necrosis factor (TNF) in sarcoidosis: who, what, and how to use them. *Sarcoidosis Vasculitis and Diffuse Lung Diseases*.

[50] Mallbris L, Ljungberg A, Hedblad MA, Larsson P, Ståhle-Bäckdahl M (2003). Progressive cutaneous sarcoidosis responding to anti-tumor necrosis factor-*α* therapy. *Journal of the American Academy of Dermatology*.

[51] Heffernan MP, Anadkat MJ (2005). Recalcitrant cutaneous sarcoidosis responding to infliximab. *Archives of Dermatology*.

[52] Heffernan MP, Smith DI (2006). Adalimumab for treatment of cutaneous sarcoidosis. *Archives of Dermatology*.

[53] Thielen AM, Barde C, Saurat JH, Laffitte E (2009). Refractory chronic cutaneous sarcoidosis responsive to dose escalation of TNF-alpha antagonists. *Dermatology*.

[54] Bachmeyer C, Blum L, Petitjean B, Kemiche F, Pertuiset E (2007). Granulomatous tattoo reaction in a patient treated with etanercept. *Journal of the European Academy of Dermatology and Venereology*.

[55] Dhaille F, Viseux V, Caudron A (2010). Cutaneous sarcoidosis occurring during anti-TNF-Alpha treatment: report of two cases. *Dermatology*.

[56] Palucka AK, Blanck JP, Bennett L, Pascual V, Banchereau J (2005). Cross-regulation of TNF and IFN-*α* in autoimmune diseases. *Proceedings of the National Academy of Sciences of the United States of America*.

[57] Cantaert T, Baeten D, Tak PP, van Baarsen LG (2010). Type I IFN and TNF*α* cross-regulation in immune-mediated inflammatory disease: basic concepts and clinical relevance. *Arthritis Research and Therapy*.

[58] Eishi Y, Suga M, Ishige I (2002). Quantitative analysis of mycobacterial and propionibacterial DNA in lymph nodes of Japanese and European patients with sarcoidosis. *Journal of Clinical Microbiology*.

[59] Bassi E, Poli F, Charachon A, Claudepierre P, Revuz J (2007). Infliximab-induced acne: report of two cases. *British Journal of Dermatology*.

[60] Korn T, Bettelli E, Oukka M, Kuchroo VK (2009). IL-17 and Th17 cells. *Annual Review of Immunology*.

[61] Facco M, Cabrelle A, Teramo A (2011). Sarcoidosis is a Th1/Th17 multisystem disorder. *Thorax*.

